# A bioconjugate vaccine against *Brucella abortus* produced by engineered *Escherichia coli*

**DOI:** 10.3389/fbioe.2023.1121074

**Published:** 2023-02-23

**Authors:** Shulei Li, Jing Huang, Kangfeng Wang, Yan Liu, Yan Guo, Xiang Li, Jun Wu, Peng Sun, Yufei Wang, Li Zhu, Hengliang Wang

**Affiliations:** ^1^ State Key Laboratory of Pathogen and Biosecurity, Beijing Institute of Biotechnology, Beijing, China; ^2^ The Third Medical Center, PLA General Hospital, Beijing, China; ^3^ Department of Clinical Laboratory, The Third Medical Centre of Chinese PLA General Hospital, The Training Site for Postgraduate of Jin Zhou Medical University, Beijing, China; ^4^ Beijing Minhai Biotechnology Co., Ltd., Beijing, China

**Keywords:** conjugate vaccines, *Brucella abortus*, bioengineering, synthetic biology, PGCT

## Abstract

Brucellosis, mainly caused by *Brucella,* is a widespread zoonotic disease worldwide, with no available effective vaccine for human use. Recently, bioconjugate vaccines against *Brucella* have been prepared in *Yersinia enterocolitica* O:9 (YeO9), whose O-antigen structure is similar to that of *Brucella abortus*. However, the pathogenicity of YeO9 still hinders the large-scale production of these bioconjugate vaccines. Here, an attractive system for the preparation of bioconjugate vaccines against *Brucella* was established in engineered *E. coli*. Briefly, the OPS gene cluster of YeO9 was modularized into five individual fragments and reassembled using synthetic biological methods through standardized interfaces, then introduced into *E. coli*. After confirming the synthesis of targeted antigenic polysaccharides, the exogenous protein glycosylation system (PglL system) was used to prepare the bioconjugate vaccines. A series of experiments were conducted to demonstrate that the bioconjugate vaccine could effectively evoke humoral immune responses and induce the production of specific antibodies against *B. abortus* A19 lipopolysaccharide. Furthermore, the bioconjugate vaccines provide protective roles in both lethal and non-lethal challenge of *B. abortus* A19 strain. Using the engineered *E. coli* as a safer chassis to prepare bioconjugate vaccines against *B. abortus* paves the way for future industrial applications.

## 1 Introduction

Brucellosis is a zoonotic infection caused by *Brucella* spp., which has a worldwide distribution. Epidemiological data demonstrate that approximately 500,000 people are infected with brucellosis annually (Mirnejad et al., 2017). *Brucella* spp.*,* including *B. abortus*, *B. melitensis*, and *B. suis,* is a family of dangerous zoonotic bacteria that can be transferred from an animal to a human host and remain pathogenic (de Figueiredo et al., 2015). Vaccination is the primary means of preventing and controlling its spread. Live attenuated vaccines, such as S19, RB51, 45/20, SR82, and Rev1, are the most common and are prepared for use in livestock (Masjedian Jezi et al., 2019). There are no licensed vaccines against *B. abortus* for human use to date. Notably, Biosafety level 3 containment and facilities are required for all culture manipulations due to the aerosol transmission of *Brucella.* Thus, it is difficult and costly for companies to develop and manufacture vaccines.

Like most Gram-negative bacteria, *Brucella* species possess lipopolysaccharide (LPS) as the most important virulence factor, which is the dominant antigen in the immune response to brucellosis (von Bargen et al., 2012). Conjugate vaccines produced by covalently linking these bacterial surface polysaccharides to proteins have shown very good protection against infections, and some have been approved for marketing, such as PREVNAR® 13 produced by Pfizer (Berti and Adamo, 2018). The classic preparation methods of conjugate vaccines involve chemical cross-linking and require the extraction and purification of bacterial polysaccharides, carrier proteins, and final cross-linked products (Berti and Adamo, 2018; Mettu et al., 2020; Morais and Suarez, 2022), leading to the relatively high cost. In addition, the extraction of antigenic polysaccharides requires the fermentation of *Brucella*, posing biosafety risks.

In recent years, with the discovery and application of bacterial protein glycosylation systems, the preparation of conjugate vaccines by Protein Glycan Coupling Technology (PGCT) has attracted increasing attention from researchers in the field of vaccine design (Harding and Feldman, 2019). In this strategy, bacterial oligosaccharyltransferases (OTase) were utilized to catalyze the transfer of bacterial antigenic polysaccharides, such as O-specific polysaccharide chains of LPS (OPS), from lipid carriers to proteins (Nothaft and Szymanski, 2010), making the vaccine preparation process easier and safer. The first reported N-linked protein glycosyltransferase is PglB from *Campylobacter jejuni*, which can be expressed in *E coli* and catalyzes the glycosylation of its natural substrate, AcrA (Linton et al., 2005). An O-linked protein glycosyltransferase PglL from *Neisseria meningitidis* was used for the preparation of bioconjugate vaccines in our team (Pan et al., 2016). In recent years, another O-linked protein glycosyltransferase, PglS, has been developed for the application of bioconjugate vaccines against *Streptococcus pneumoniae* (Harding et al., 2019).

Regarding PGCT technology, ensuring the O antigen synthesis gene cluster of the target bacteria is functional in the exogenous host cell is crucial. At present, no reports demonstrate the successful expression of the O antigen synthesis gene cluster of *Brucella* in *E coli*. Fortunately, through serological analysis and NMR identification, a high degree of similarity has been found between the O antigen of *B. abortus* and that of YeO9, both of which use the same monosaccharide N-formyl-perosamine as the basic module (Bundle et al., 1989; Skurnik et al., 2007). For this reason, YeO9 have been used as an engineered host to prepare a glycosylated AcrA protein bearing the antigenic polysaccharides of *B. abortus* using an N-linked PGCT system. This protein can react with serum against *B. abortus* but lacks sufficient protective effects in challenge experiments (Iwashkiw et al., 2012). Inspired by this work, our team optimized the design of this vaccine and produced a bioconjugate vaccine for *B. abortus* with cholera toxin B subunit (CTB) as the carrier protein using an O-linked PGCT system in YeO9. Animal experiments showed that the vaccine provided promising levels of protection (Huang et al., 2020). However, as YeO9 is an opportunistic pathogen, the risk for zoonotic disease remains (Galindo et al., 2011; Le Guern et al., 2016; Rivas et al., 2021).

Using engineered *E. coli* as chassis cells to prepare bioconjugate vaccines is a feasible pathway to solve the biosafety issues involved in the development of effective vaccines against highly pathogenic organisms (Dow et al., 2020). Given that the activity of the O antigen synthesis gene cluster remains unclear in *B. abortus*, the YeO9 antigen synthesis gene cluster was also used to produce conjugate vaccine in engineered *E coli* with the help of our O-linked PGCT system (PglL system). Fortunately, the desired glycoproteins with pentamer CTB as the carrier protein were successfully produced by *E. coli*, and a series of experiments demonstrated that this bioconjugate vaccine is safe and effective; the vaccine could protect mice in challenge experiments with different doses of *B. abortus* A19.

## 2 Methods and materials

### 2.1 Bacterial strains, plasmids, primers, and growth conditions

The strains and plasmids used in this study are listed in Supplementary Table S1, and the primers are listed in Supplementary Table S2. All strains of the *E. coli* W3110 series were cultured in Luria-Bertani (LB) liquid medium or LB medium containing 1.5% agarose at 37°C; YeO9 was cultured in Brain Heart Infusion (BHI) medium containing 1.5% agarose at 30°C; and *B. abortus* A19 was cultured in Tryptic Soy Broth (TSB) medium or TSB medium containing 1.5% agarose at 37°C. For protein expression, the strains carrying expression plasmids were cultured at 37°C to an OD_600 nm_ of approximately 0.6. Then, 1 mM isopropyl-β-D-thiogalactopyranoside (IPTG) was added at 30°C for 12 h.

### 2.2 Experimental animals

Seven-week-old female BALB/c mice (free of specific pathogens) were used in this study and purchased from Beijing Vital River Laboratory Animal Technology Co., Ltd. All immunization experiments were performed following ethical regulations for animal testing and research. All experiments were approved by and conducted in accordance with the guidelines of the Academy of Military Medical Sciences Institutional Animal Care and Use Committee (approval code: IACUC-DWZX-2021-008).

### 2.3 LPS and OPS extraction

LPS extraction was performed as described previously (Sun et al., 2018). Briefly, the cells were collected by centrifugation and resuspended in ddH_2_O. Then, an equal volume of 90% phenol was added, and the mixture was shaken vigorously at 68°C for 30 min. After centrifuging at 7,000 *g* for 20 min at 4°C, the supernatant was collected. Phenol was removed from the supernatant using a dialysis bag in ddH_2_O for 2 days. Then, DNase (5 μg/mL; Solarbio, Beijing, China), RNase (1 μg/mL; Solarbio) and proteinase K (20 μg/mL; Solarbio) were sequentially added to the dialyzed sample. After incubating at the optimal temperature, the solution was placed in a boiling water bath for 10 min and then centrifuged at 7,000 *g* for 10 min to obtain LPS. To obtain OPS, glacial acetic acid was added to the LPS solution with a final concentration of 1% and incubated in a boiling water bath for 90 min. The pH was then adjusted to 7.0 with NaOH. Finally, the mixture was centrifuged at 40,000 *g* for 5 h, and the supernatant was collected.

### 2.4 Purification of target glycoproteins

Cells were collected by centrifuging at 8,000 *g* for 10 min at 4°C and then resuspended in A1 (20 mM Tris-HCl pH 7.5, 10 mM imidazole, 500 mM NaCl). Then, the cells were lysed using a homogenizer and centrifuged to collect the supernatant, which was subsequently was applied to a chelating column (Complete His-Tag Purification Resin, Roche, Penzberg, Germany). After washing with A1, bound protein was eluted with B1 (20 mM Tris-HCl pH 7.5, 500 mM imidazole, 500 mM NaCl). Then, the sample was further purified using a Sephadex 200 (GE Healthcare) column. The fractions were collected and analyzed using SDS-PAGE.

### 2.5 Western blot analyses

Western blotting was performed, as described previously (Pan et al., 2016). Horseradish peroxidase (HRP)-conjugated 6×His-tag antibody (Abmart, Shanghai, China) (1:3,000) was used to detect 6×His-tag-fused proteins. Monoclonal *Yersinia enterocolitica* O:9 antibody (Fitzgerald, Acton, MA) (1:400) and *Brucella* antibody (1:400) to detect glycoproteins. The antibody against *Brucella* was produced by immunizing rabbits with whole *Brucella suis* S2 and blocking with *E. coli* W3110 cell lysates. HRP-conjugated anti-rabbit IgG (TransGen Biotech, Beijing, China) (1:15,000) was used as a secondary antibody.

### 2.6 Monosialic acid tetrahexose ganglioside (GM1) binding assay

The GM1 binding assay was performed as described previously (Li et al., 2022). Briefly, a 96-well plate was coated with 100 µL of GM1 solution (Sigma; 2 μg/mL) overnight at 4°C. After washing with PBST (PBS containing 0.05% Tween), 200 µL of blocking solution (5% skim milk in PBST) was added to each well. After incubating at 37°C for 2 h, the plate was washed again. Then, 100 µL of samples at different dilutions were added and incubated at 37°C for 1 h. After washing again, 100 µL of anti-CTB antibody was added to each well and incubated at 37°C for 1 h. After another washing step, 100 µL of HRP-labeled goat anti-rabbit antibody (1:5,000) was added and incubated at 37°C for 1 h. The plate was washed again and the soluble TMB Kit (CWbio, Beijing, China) was used for color development. The absorbance at a wavelength of 450 nm was measured using a microplate spectrophotometer.

### 2.7 Flow cytometric analysis of mouse spleen cells

The mice were humanely sacrificed, and the spleens were removed. The spleens were triturated, and the red blood cells were removed using RBC Lysis Buffer (Solarbio) according to the kit instructions. After centrifuging at 500 *g* for 10 min at 4°C, the supernatants were discarded, and cells were resuspended and washed twice with Staining buffer (eBioscience) to obtain single-cell suspensions. Then, cells were stained with different combinations of flow cytometry antibodies, including APC-conjugated anti-mouse CD3 (eBioscience, San Diego, United States), FITC-conjugated anti-mouse CD4 (BioLegend, San Diego, United States), PE-conjugated anti-mouse CD8 (eBioscience) APC-conjugated anti-mouse B220 (BioLegend), Pacific Blue-conjugated anti-mouse GL-7 (BioLegend), PE-conjugated anti-mouse CD95 (BioLegend), PE-conjugated anti-mouse PD-1 (BioLegend), and Brilliant Violet 421-conjugated anti-mouse CXCR5 (BioLegend). After staining, cells were washed with Staining buffer and dispersed in 500 mL of staining buffer. Analysis was performed using a Mona CytoFLEX flow cytometer (BeckmanCoulter LifeSciences, Brea, United States).

### 2.8 Animal immunization and challenge

BALB/c mice were intraperitoneally injected with the vaccine formulation, including CTB-OPS_Ba_ (2.5 µg polysaccharide), OPS_Ba_ (2.5 µg), CTB, and PBS, on days 0, 14, and 28. Seven days after each injection, blood was collected from the tail vein of each mouse, and the serum was isolated. The bacteria were cultured at 37°C in TSB medium. When the OD600 reached 2.0, the bacteria were diluted to the required concentration with saline. Then, the diluted bacteria were injected intraperitoneally into the mice (200 µL/mouse).

### 2.9 ELISA

First, the 96-well plates were coated with 100 µL of *B. abortus* A19 LPS (100 μg/mL) and incubated overnight at 4°C. Then, the plates were washed with PBST 3 times, and 200 μL of blocking buffer (5% skim milk in PBST) was added to each well and incubated at 37°C for 2 h. After blocking, sera from each mouse were added to the corresponding wells and serially diluted with dilution buffer (10% blocking buffer) and incubated at 37°C for 1 h. The plates were washed and 100 μL of HRP-conjugated goat anti-mouse IgG, IgG1, IgG2a, IgG2b, or IgG3 antibody (Abcam, Cambridge, MA, United States) (1:15,000) was added to each well and incubated at 37°C for 1 h. After washing, the Soluble TMB Kit (CWBio, Beijing, China) was used for color development, and the absorption at a wavelength of 450 nm was measured using a microplate spectrophotometer.

### 2.10 Cytokine detection

The cytokine levels were detected with the Double Antibody Sandwich ELISA method using cytokine detection kits (Dakowei Medical Equipment Co., Ltd., Shenzhen, China). Briefly, 100 μL of serum samples and standards at different dilutions were added to pre-coated wells, then 50 μL of Biotinylated antibodies (1:100) were added to each well. After incubating at 37°C for 90 min, the plates were washed 3 times and dried. Then, 100 μL of Streptavidin-HRP (1:100) was added to each well and incubated at 37°C for 30 min. After washing and drying again, TMB solution (100 μL/well) was added and incubated at 37°C for 15 min in the dark. The reaction was then terminated by adding Stop solution (100 μL/well). The absorbance in each well at a wavelength of 450 nm was measured using a microplate spectrophotometer.

### 2.11 Spleen bacterial load assay

Fourteen days after the third immunization, 2.81 × 10^7^ CFU of *B. abortus* A19 strain was intraperitoneally injected into each mouse. The control group was injected with the same volume of PBS. On the 7th day after infection, the mice were humanely sacrificed, and the spleens were removed. The spleens were weighed and then grind in 1 mL of sterile saline. The grind spleen samples were then serially diluted with sterile saline, plated on TSA solid medium, and incubated at 37°C for 72 h. Subsequently, the bacterial colonies were counted.

### 2.12 Statistical analysis

The data are presented as the mean ± SD. Statistical analyses were performed using GraphPad Prism 8.0 (GraphPad, San Diego, CA, United States). Data were analyzed *via* one-way ANOVA with Dunnett’s multiple comparison test for multiple-group comparisons. A log-rank test was used for the survival analysis. *p* values below 0.05 indicated significance (*p < 0.05, **p < 0.01, ***p < 0.001, ****p < 0.0001).

## 3 Results

### 3.1 Construction of the YeO9 OPS synthesis plasmid and its functional verification in *E coli*

**FIGURE 1:**
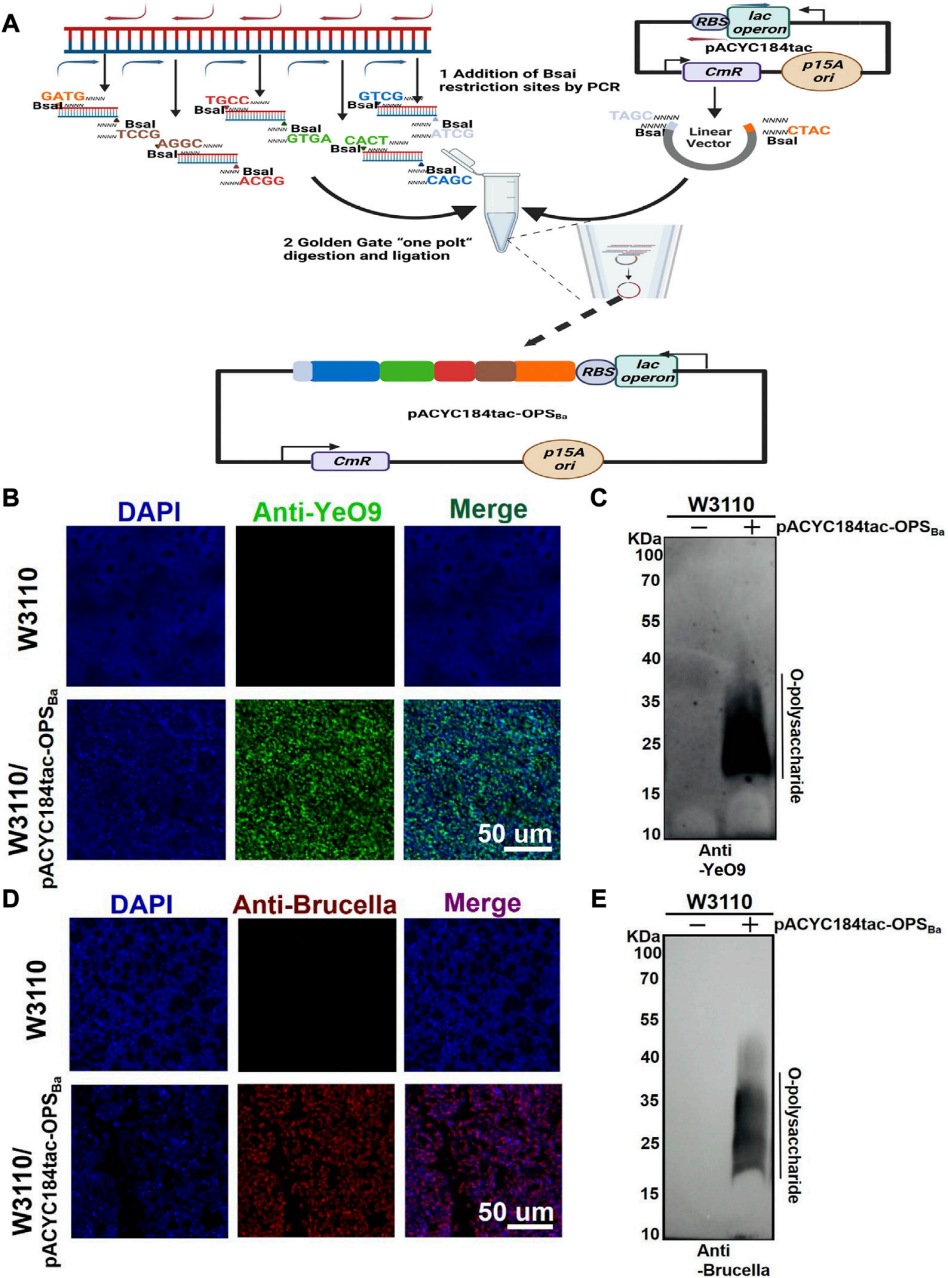
Synthesis of YeO9 OPS in *Escherichia coli*. **(A)** Schematic diagram of YeO9 OPS synthesis plasmid construction with the Golden Gate cloning method. **(B)** Fluorescence imaging analysis of the reaction between *Escherichia coli* W3110/pACYC184tac-OPS_Ba_ and the YeO9 antibody. **(C)** W3110/pACYC184tac-OPS_Ba_ LPS was detected with YeO9 serum by Western blotting. **(D)** Fluorescence imaging analysis of the reaction between *Escherichia coli* W3110/pACYC184tac-OPS_Ba_ and *Brucella* serum. **(E)** W3110/pACYC184tac-OPS_Ba_ LPS was detected with *Brucella* serum by Western blotting.

YeO9 OPS was first synthesized in *E. coli* by constructing an appropriate plasmid. Considering that the gene cluster of YeO9 OPS is approximately 15,000 bp in length and comprises 12 genes (*manC-wbcW*) (Supplementary Figure S1), we planned to connect it to a plasmid backbone using the Golden Gate cloning method. Briefly, the OPS gene cluster was divided into five fragments, and these fragments and the pACYC184tac vector were linearized by PCR. All the fragments were designed with unique overhangs at each end. Then, the fragments were ligated to the vector skeleton using the Golden Gate Assembly Kit (BsaI-HFv2) through one-step method and the new plasmid was named pACYC184tac-OPS_Ba_ (Figure 1A).

To verify the synthesis of YeO9 OPS in *E. coli*, we first transferred pACYC184tac-OPS_Ba_ into *E. coli* W3110, which cannot independently synthesize O-polysaccharide. After culturing in LB medium at 37°C overnight, the cells were successively incubated with YeO9 antibody and fluorescent-labeled secondary antibody. Through fluorescence imaging, we found that the Anti-YeO9 signal was obviously present in *E. coli* W3110 containing pACYC184tac-OPS_Ba_, while no visible signal was found in the untreated W3110 strain (Figure 1B). Then, LPS was extracted, and the immunoblotting assay results also showed that the synthesized OPS could react with YeO9 antibody (Figure 1C). Furthermore, we used *Brucella* serum to verify its cross-reactivity. As expected, the fluorescence imaging results showed that the heterologously synthesized OPS cross-reacted with *Brucella* serum (Figure 1D). Similarly, immunoblotting results were consistent with the fluorescence imaging results (Figure 1E).

### 3.2 Biosynthesis of *B. abortus* conjugate vaccine in engineered *E. coli*

**FIGURE 2:**
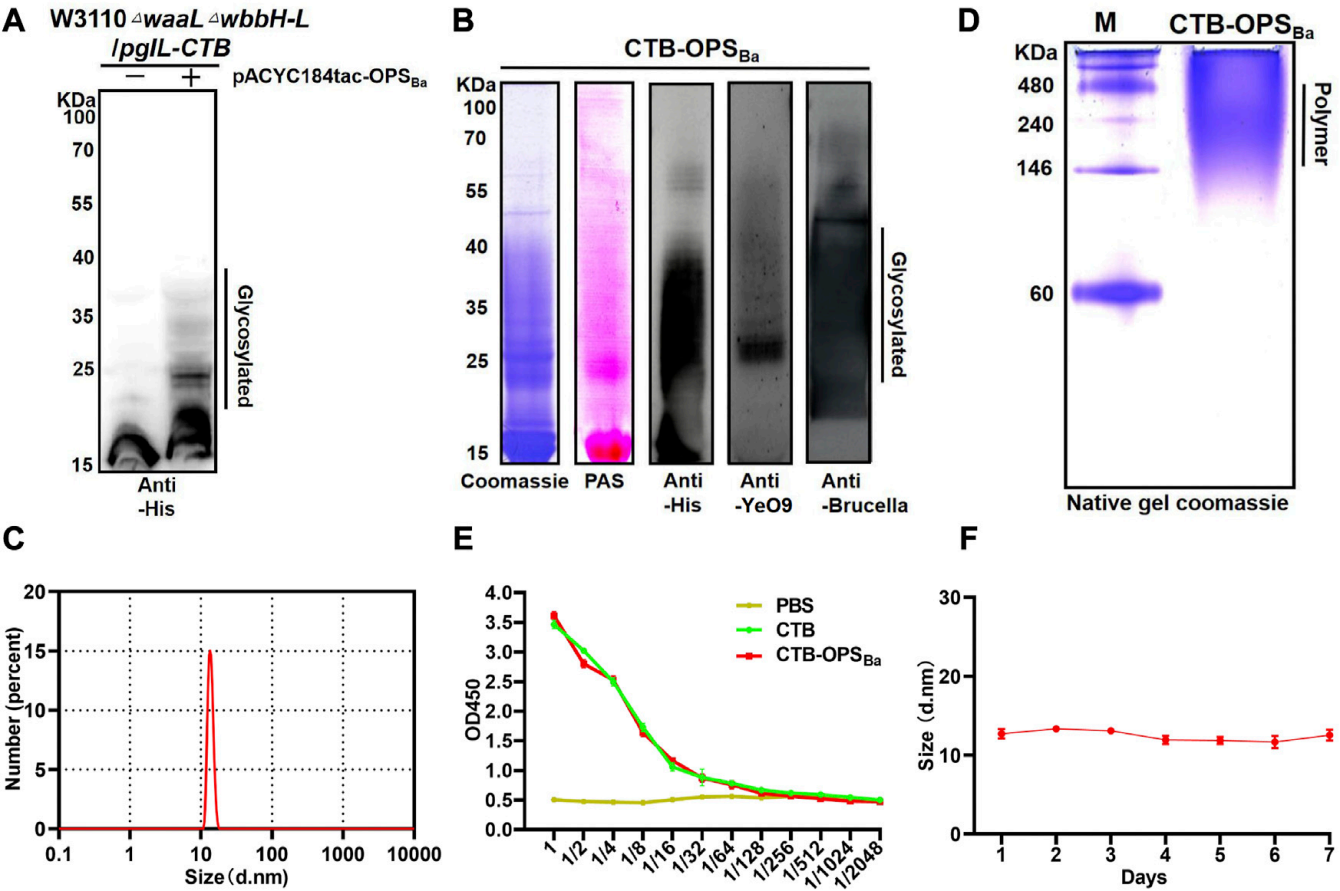
Expression and Verification of the glycoprotein. **(A)** PgIL-mediated protein O-glycosylation in W3110 △*waal*△*wbbH-L* strain was detected by Western blotting with 6 × His Tag antibody. **(B)** Purified CTB-OPS_Ba_ samples were separated by SDS-PAGE and analyzed by Coomassie blue staining, PAS staining and Western blotting with 6 × His Tag antibody, YeO9 antibody, and *Brucella* serum. **(C)** Dynamic light scattering analysis of CTB-OPS_Ba_. **(D)** CTB-OPS_Ba_ was separated by non-denaturing electrophoresis and stained by Coomassie blue. **(E)** ELISA was performed to analyze the GM1 binding ability of CTB-OPS_Ba_. **(F)** Stability analysis of CTB-OPS_Ba_ measured by dynamic light scattering.

After confirming the successful synthesis of the heterologous OPS in *E. coli* W3110 and determining its specificity, we further transformed pACYC184tac-OPS_Ba_ into our previously constructed strain W3110Δ*waal*Δ*wbbH-L*, which can block the transformation of OPS to lipid A and is more suitable for exogenous polysaccharide synthesis. The plasmid pET28a-*pgIL-CTB*4573 co-expressing glycosyltransferase PglL and CTB with glycosylation sequence 4,573 was also transformed into W3110Δ*waal*Δ*wbbH-L* to couple the carrier protein with the OPS. After inducing with IPTG, the whole bacterial sample was separated by SDS-PAGE, and Western blotting with a 6 × His tag antibody was performed to detect the glycosylation of carrier proteins. The result showed a typical glycosylation ladder band when the polysaccharide gene cluster was co-expressed with the pglL and CTB, while only the carrier protein band was detected when the polysaccharide gene cluster was not co-expressed. This result suggests that OPS can be successfully coupled with CTB to generate glycoprotein (CTB-OPS_Ba_) *via* the catalysis of glycosyltransferase PglL (Figure 2A). Then, the heterologously synthesized glycoprotein was purified *via* affinity and size-exclusion chromatography, and a series of verifications were performed. Coomassie blue staining and periodic acid Schiff staining showed consistent bands compared to the previous whole-cell immunoblot. In addition, the glycoprotein was detected by Western blotting using an anti-6 × His-Tag antibody, an anti-YeO9, and *Brucella* serum sequentially, showing that the CTB-OPS_Ba_ could react with both YeO9 antibody and *Brucella* serum (Figure 2B).

CTB is known to form a pentamer in natural conditions and is often used as an immune activator because of its ability to bind with GM1, which is widely present on the surface of antigen-presenting cells (Beddoe et al., 2010). Moreover, the formation of a pentamer is a necessary condition for GM1 binding. Thus, we analyzed the particle size of CTB-OPS_Ba_ by dynamic light scattering (DLS) and the results showed that the CTB-OPS_Ba_ was approximately 13 nm in diameter (Figure 2D). A native PAGE was also performed, and the Coomassie blue staining results showed that the molecular weight of the CTB-OPS_Ba_ glycoprotein was approximately 224–250 kDa, which is approximately 5-fold that of the monomeric protein molecular weight (Figure 2C), indicating that glycosylation doesn’t affect CTB polymerization. Further, we determined the binding capability of CTB-OPS_Ba_ with GM1 by ELISA method using the CTB standard and PBS as control. The results showed that the OD_450_ values decreased as the dilution of the CTB-OPS_Ba_ and CTB standard increased (Figure 2E), indicating that CTB retained the ability to bind to receptors after glycosylation. Moreover, to evaluate the stability of the glycoproteins, we examined the size and molecular weight of CTB-OPS_Ba_ at different time points at room temperature. The results showed no significant size change or obvious degradation even after 7 days (Figure 2F; Supplementary Figure S3), suggesting that the purified CTB-OPS_Ba_ was stable and could be stored at room temperature for a long time.

### 3.3 Immune activation and splenic stimulation of bioconjugate vaccines

**FIGURE 3:**
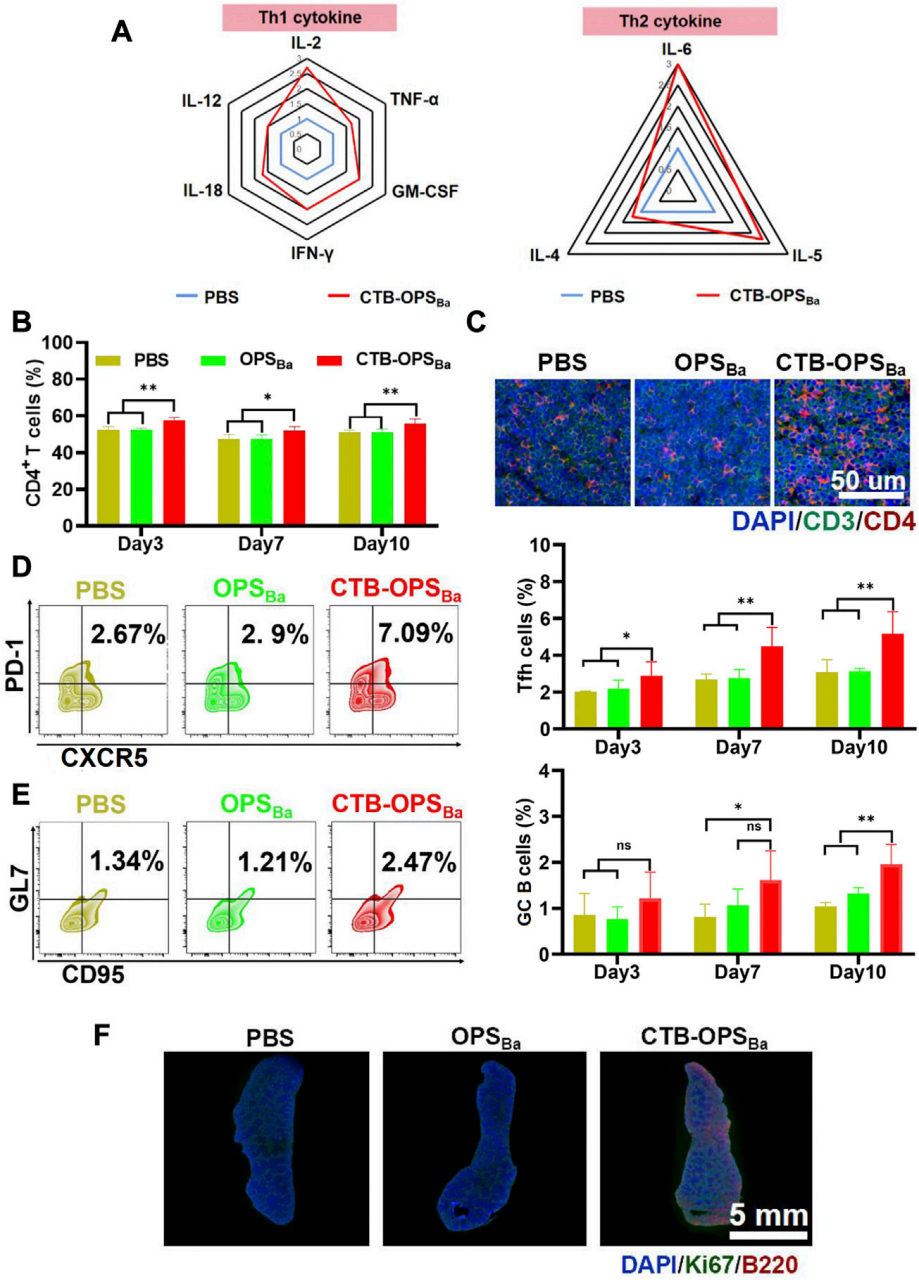
Evaluation of the immune response induced by CTB-OPS_Ba_. **(A)** Th1- and Th2-related cytokines in serum were measured 6 h after immunization. **(B)** CD4^+^ T cells in spleens were detected by flow cytometry on days 3, 7, and 10 after immunization. **(C)** CD3^+^ and CD4^+^ cells in spleens were detected by immunofluorescence on days 3, 7, and 10 after immunization. Tfh cells (CXCR5^+^ and PD-1^+^ in the CD4^+^ cell population) **(D)** and GC B cells (GL7^+^ and CD95^+^ in the B220^+^ cell population) **(E)** in spleens were detected by flow cytometry. **(F)** Ki67 and B220 were detected in spleens on the 10th day after immunization by immunofluorescence. n = 5, and all data are expressed as the mean ± SD. One-way ANOVA with Dunnett’s multiple comparison test was used to compare CTB-OPS_Ba_ group data with the data from the other groups: **p < 0.01, *p < 0.05, ns *p* > 0.05.

Immune cells (e.g., APCs) recognize and present antigens and stimulate differentiation of CD4^+^ T cells, resulting in a Th1 and Th2 immune response. BALB/c mice were immunized with CTB-OPS_Ba_ and PBS, and peripheral blood was collected after 6 h. Serum cytokine levels were measured through the LiquiChip method, and the results showed increased concentrations of many Th1- and Th2-related cytokines (Figure 3A). We further examined CD4^+^ T cells in the spleens by flow cytometry at 3, 7, and 10 days after immunization, and the results showed that the spleens in CTB-OPS_Ba_-treated mice had the highest percentage of CD4^+^ T cells at all three time points (Figure 3B). This result was also confirmed by the mouse spleen cell surface fluorescent antibody assay, which showed more CD3^+^ and CD4^+^ cells in the spleen sections (Figure 3C; Supplementary Figure S4).

The germinal center is the main site of the thymus-dependent antigen response and is formed approximately 1 week after antigen stimulation. The main function of follicular T cells (Tfh cells) is to help B cells differentiate into effector cells in the germinal center. To verify the effect of CTB-OPS_Ba_ on Tfh cells and germinal center B cells, we first examined the changes in Tfh cells in the spleen of mice on days 3, 7, and 10. The flow cytometric results showed that CTB-OPS_Ba_ increased the proportion of these cells in mouse spleens and that the cell proportion increased as the days of antigen stimulation increased (Figure 3D). The changes in germinal center B cells were consistent with those in Tfh cells, and CTB-OPS_Ba_ could increase the percentage of germinal center B cells (Figure 3E). Additionally, the Ki67/B220 fluorescence results on day 10 showed significant splenic B-cell proliferation in the CTB-OPS_Ba_ group, while no B-cell proliferation was found in the PBS and OPS groups (Figure 3F; Supplementary Figure S5), suggesting that CTB-OPS_Ba_ stimulated the proliferation of mouse spleen germinal center B cells, leading to the activation of more B cells. The above results indicate that CTB-OPS_Ba_ can effectively stimulate the proliferation of mouse spleen lymphocytes and the humoral immune response.

### 3.4 Safety evaluation of bioconjugate vaccine

**FIGURE 4:**
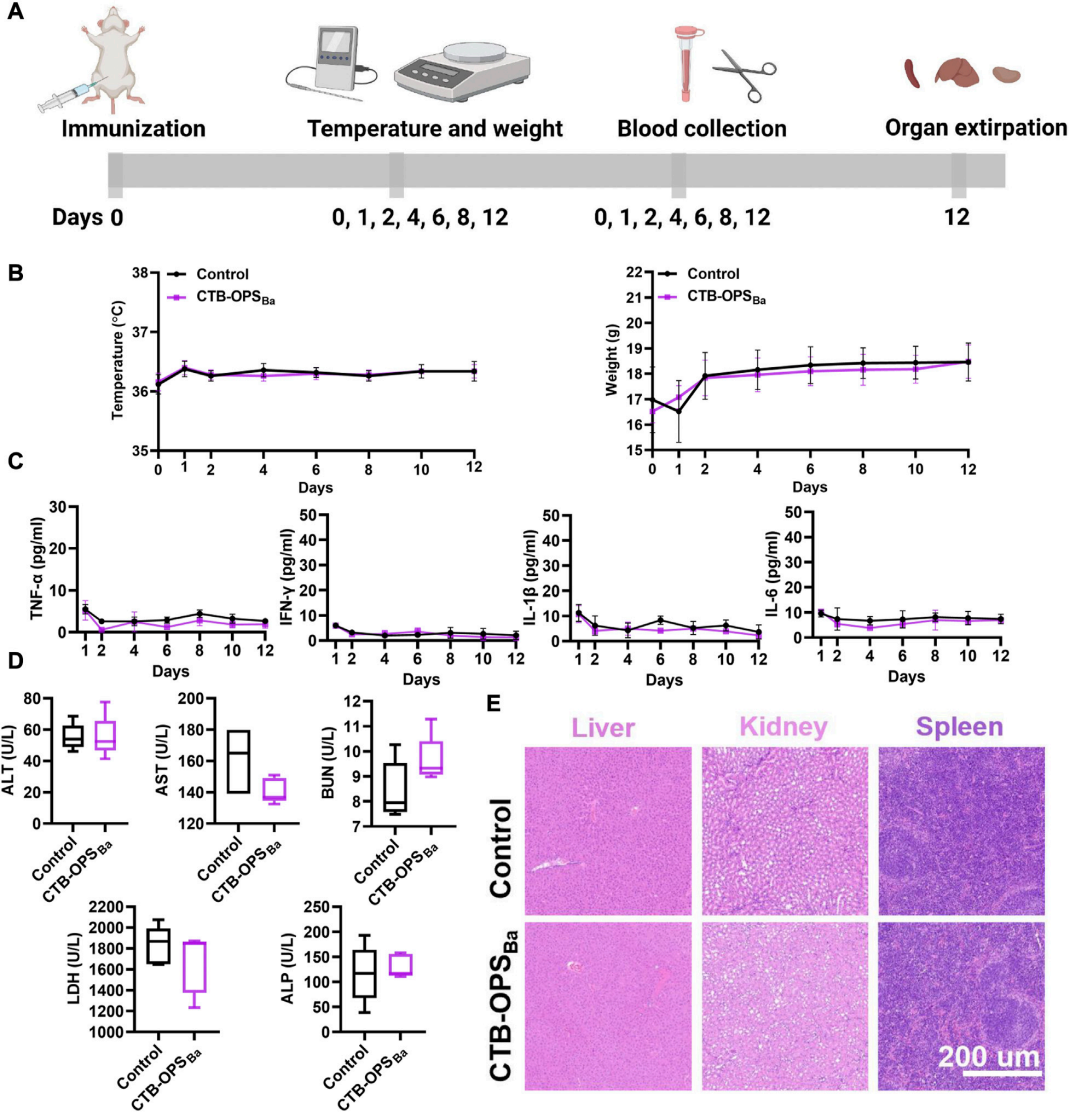
Safety evaluation of *B. abortus* candidate bioconjugate vaccine (n = 5). **(A)** Overall procedure of vaccine safety evaluation. **(B)** Body temperature and body weight changes of mice during the observation period. **(C)** Cytokines (including TNF-α, IL-6, IL-1β, and IFN-γ) in the serum at different time points. **(D)** Biochemical indices (including ALT, AST, BUN, LDH, and ALP) in the serum 12 days after immunization. **(E)** HE staining analysis of mouse liver, spleen, and kidney samples.

We evaluated the safety of the CTB-OPS_Ba_ before proceeding to the mouse immune study. After injecting CTB-OPS_Ba_ (25 μg polysaccharide, 10 times the normal dose) into each mouse, a series of indicators, including body temperature, body weight, serum cytokines, serum biochemical indices, and tissue and organ pathology were tested during the observation period. Untreated mice were used as a control (Figure 4A). Within 12 days of vaccination, both groups of mice maintained normal feeding habits, with similar changes in body temperature and weight (Figure 4B). Meanwhile, we examined serum inflammatory factors, such as TNF-α, IL-6, IL-1β, and IFN-γ, in the mice during the 12-day period. As shown in Figure 4C, these inflammatory factors were maintained at a very low level in all mice, with no significant difference between the two groups. On the 12th day after immunization, we measured five biochemical indices (ALT, AST, BUN, LDH, and ALP) in the serum of each mouse, and all were within the normal range (Figure 4D). Furthermore, the spleen, liver and kidney were collected from each mouse, and the HE staining results showed no damage in the CTB-OPS_Ba_ treated mice (Figure 4E). These results indicate that CTB-OPS_Ba_ prepared in engineered *E. coli* is safe and can be evaluated in subsequent animal experiments.

### 3.5 Evaluation of specific antibodies after immunization with the bioconjugate vaccine

**FIGURE 5:**
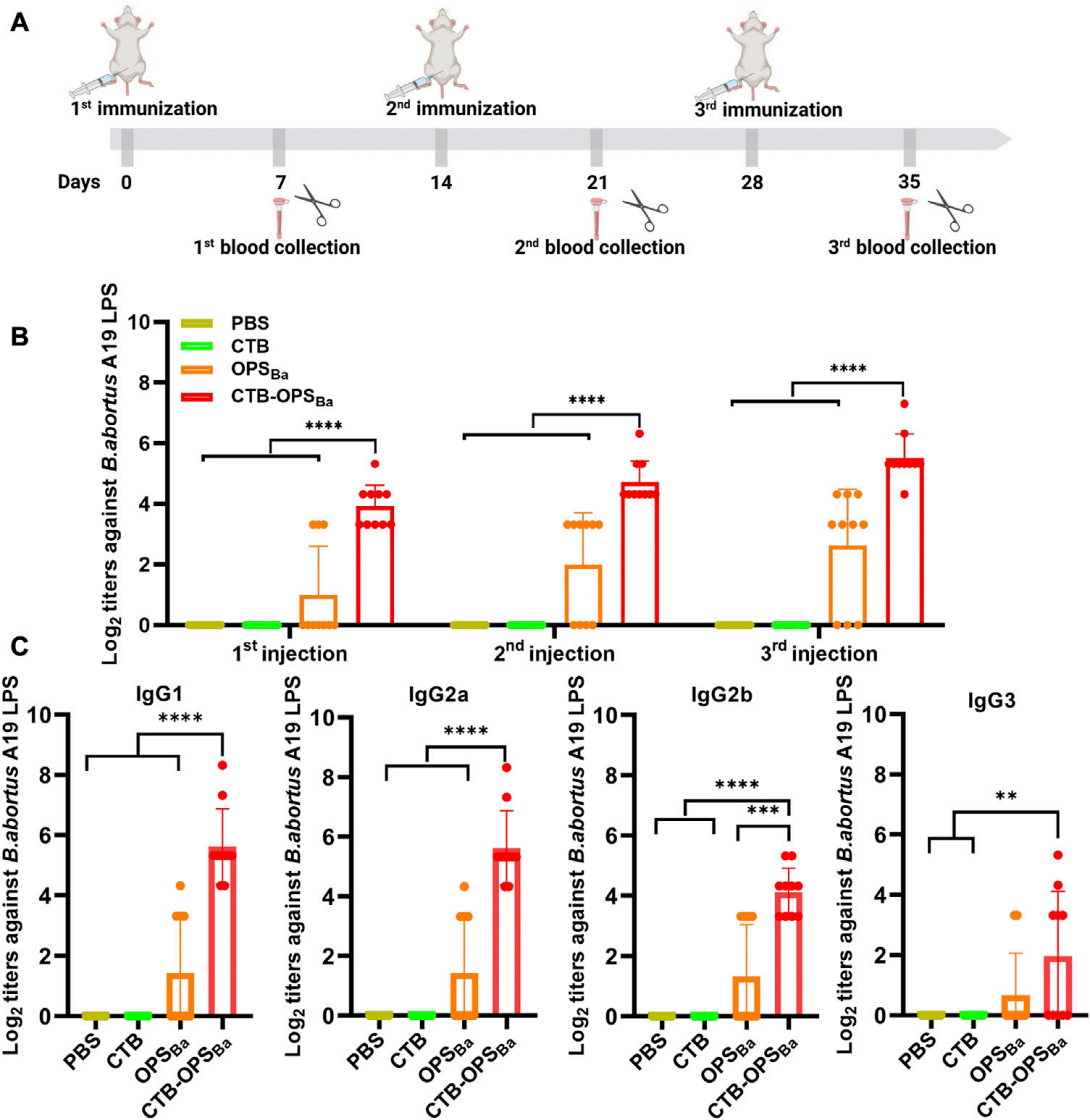
Specific antibody response induced by CTB-OPS_Ba_. **(A)** Immunization schedule for further evaluation. **(B)** On the 7th day after immunization, the IgG antibody titer against *B. abortus* A19 LPS was measured by ELISA. **(C)** Seven days after the third immunization, the IgG subtype antibody titers (IgG1, IgG2a, IgG2b, and IgG3) against *B. abortus* A19 LPS were measured. n = 10, and all data are presented as the mean ± SD; one-way ANOVA with Dunnett’s multiple comparison test was used for comparisons: ****p < 0.0001, ***p < 0.001, **p < 0.01, ns *p* > 0.05.

To verify whether the CTB-OPS_Ba_ could induce specific antibodies against *B. abortus*, BALB/c mice were immunized intraperitoneally with OPS_Ba_ (2.5 µg), CTB-OPS_Ba_ (2.5 µg polysaccharide), CTB alone, and PBS on day 0, 14, and 28. Seven days after each immunization, blood was collected from the tail vein. The levels of IgG antibodies and antibodies against *B. abortus* A19 LPS in the sera were measured by ELISA (Figure 5A). The results showed that both OPS_Ba_ and CTB-OPS_Ba_ could induce specific antibodies after each immunization, and the titer in the CTB-OPS_Ba_ group was significantly higher. Meanwhile, no specific antibodies were observed in the PBS and CTB groups (Figure 5B). Further, we evaluated IgG subtypes (including IgG1, IgG2a, IgG2b, and IgG3) against *B. abortus* A19 LPS after the third immunization. The CTB-OPS_Ba_ group showed the highest antibody titer for all subtypes (Figure 5C). The level of IgG1 and 3 and IgG2a and 2b represent the intensity of the Th2 and Th1 immune responses, respectively; thus, CTB as a carrier can enhance the immune response, consistent with the results of previous studies.

### 3.6 Evaluation of bioconjugate vaccine-induced protection after different doses of *B. abortus* A19 infection in mice

**FIGURE 6:**
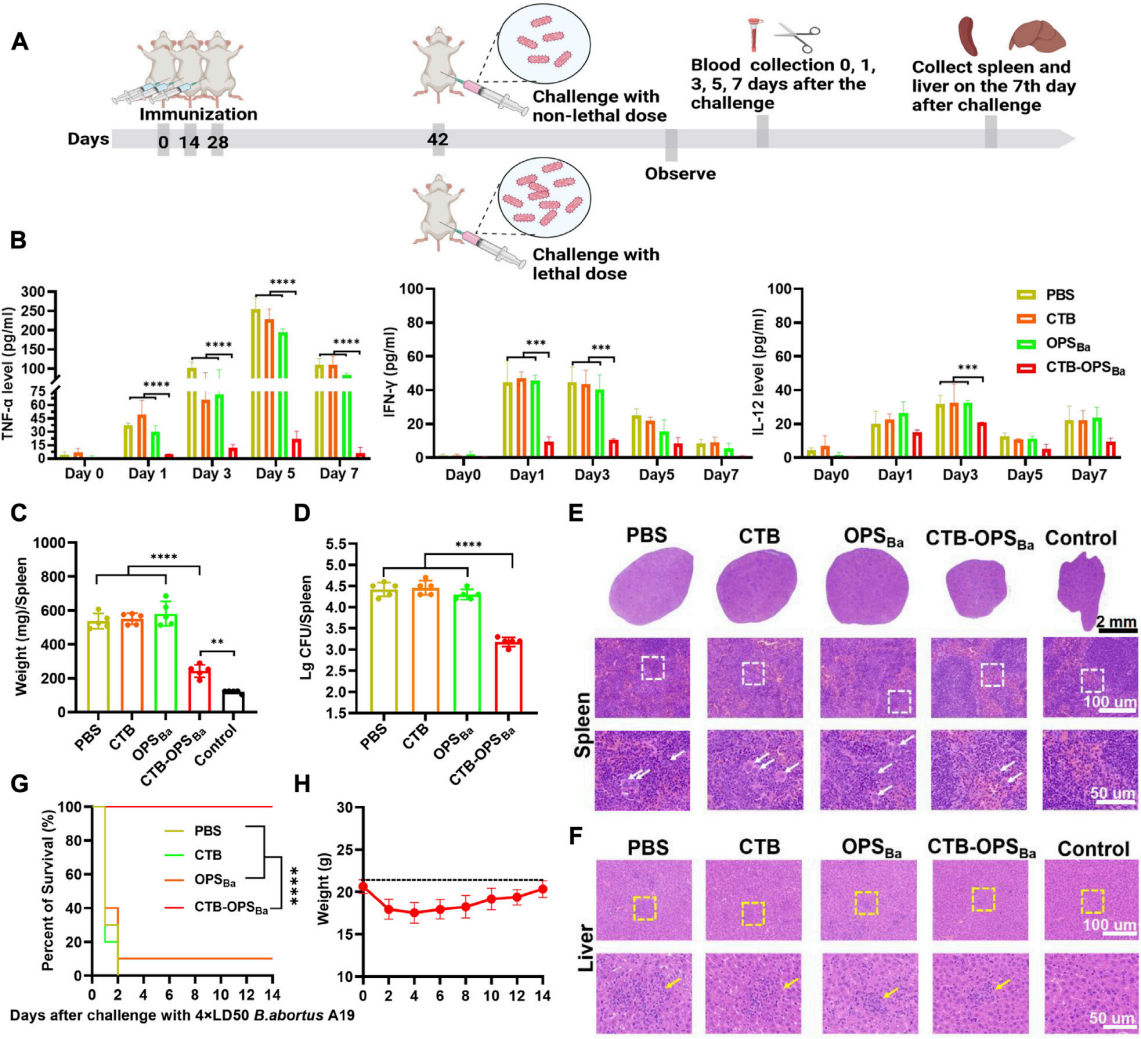
Evaluation of CTB-OPS_Ba_-mediated protection against different doses of *B. abortus* A19. **(A)** Schematic diagram of the establishment of the infection model and subsequent evaluations. **(B)** After infection with 2.81 × 10^7^ CFU *B abortus* A19 per mouse, tail vein blood was collected on days 0, 1, 3, 5, and 7, and the levels of TNF-α, IFN-γ and IL-12 in the sera were measured by ELISA (n = 10). **(C–D)** On the 7th day after infection with 2.81 × 10^7^ CFU *B abortus* A19 per mouse, the spleens of each mouse were weighed **(C)** and the bacterial load in the spleens was detected (n = 5) **(D)**. **(E)** Spleens were also stained with HE at the 7-day time point as shown in the lower whited-frame image; the white arrows represent multinucleated giant cells. **(F)** On the 7th day after infection, the livers from each mouse were analyzed by HE staining as shown in the yellow-framed images; the yellow circles indicate lymphoproliferative nodules. **(G)** Mice were infected with 2.1 × 10^8^ CFU *B abortus* A19 per mouse and their survival rates were measured (n = 10). **(H)** The body weight changes of the mice in the CTB-OPS_Ba_ group within 14 days were recorded. All data are expressed as the mean ± SD. One-way ANOVA and Dunnett’s multiple comparison test was used for comparisons: ****p < 0.0001, ***p < 0.001, **p < 0.01. Survival was calculated using a log-rank test:****p < 0.0001.

To further examine the protective effect of CTB-OPS_Ba_, the immunized mice were infected with non-lethal and lethal doses of the *B. abortus* A19 strain, and a series of protective indicators were evaluated (Figure 6A). First, on day 14, after three immunizations, we intraperitoneally injected each group of mice with 2.81 × 10^7^ CFU *B abortus* A19 per mouse to detect the changes in cytokines (TNF-α, IFN-γ, IL-12) after infection. The three inflammatory evidence factors in CTB-OPS_Ba_-immunized mice remained at low levels at all observed time points (*p* < 0.001), while the other groups showed a significant increase after infection, especially in TNF-α, which reached 250 pg/mL in the PBS and CTB groups (Figure 6B).

*Brucella* infestation mainly colonizes the spleen and the liver, causing a series of associated pathologies. Thus, we collected the spleens of mice on day 7 after infection for weighing, bacterial load assays, and HE staining. These results showed that all mice infected with *B. abortus* A19 had altered spleen indicators compared to the control group. Among them, the CTB-OPS_Ba_ group showed the smallest change in spleen weight (Figure 6C; Supplementary Figure S6). Similarly, the CTB-OPS_Ba_ group had the lowest spleen bacterial load of approximately 10^3^–10^3.3^, which was statistically significant compared to that of the other groups (Figure 6D). HE staining of the spleens of CTB-OPS_Ba_ immunized mice revealed that these spleens demonstrated a more pronounced red-white bone marrow border, fewer multinucleated giant cells, and a better-maintained lymphocyte ratio than the spleens from mice in the other groups (Figure 6E). We also performed HE staining of the liver; as expected, the pathological changes in the liver were consistent with those in the spleen (Figure 6F).

Finally, after three immunizations, we injected *B. abortus* A19 (2.1 × 10^8^ CFU/mouse (4 × LD50) (Huang et al., 2020)) intraperitoneally into mice to assess the protective effect of CTB-OPS_Ba_ on mice infected with lethal doses. Within 14 days after infection, we observed the survival rate of the mice in each group and recorded the body weight changes of the mice in the CTB-OPS_Ba_ group. The results showed that all mice in the PBS and CTB groups died on the third day after infection. The survival rate of mice in the OPS_Ba_ group was approximately 10%, while the survival rate of mice in the CTB-OPS_Ba_ group was 100% (Figure 6G). Mice in the CTB-OPS_Ba_ group showed a decreasing trend in body weight during the first 3 days after infection (approximately 3 g) and gradually recovered on the sixth day (Figure 6H). This result indicated that vaccination of the CTB-OPS_Ba_ group could provide excellent protection against *B. abortus* infection.

## 4 Discussion

Currently, there are two practicable strategies for preparing bioconjugate vaccines using PGCT methods. One is to engineer the targeted pathogenic bacteria into the host strains by knocking out the virulence genes. The advantage of this strategy is that there is no need to clone large fragments of the gene cluster to synthesize antigenic polysaccharides. However, the cultivation conditions of attenuated strains should be optimized in the process of large-scale preparation, and some strains might pose certain biosafety risks. Another method, as used in this study, is to produce conjugate vaccines using engineered *E. coli*. Many reports have shown that *E coli* can be used to produce bioconjugate vaccines against pathogens such as *Shigella dysenteriae*, *Francisella tularensis*, *Burkholderia pseudomallei*, *E. coli O157:H7, S. pneumoniae* (Kay et al., 2016)*,* and *Klebsiella pneumoniae* (Dow et al., 2020). The key technical step of this method is obtaining the O antigen synthesis gene cluster, commonly as large DNA fragments that are difficult to clone accurately due to the spontaneous mutations that occur during PCR. The rapid development of synthetic biology techniques has led to the development of novel DNA assembly technologies. For example, the Golden Gate cloning technology used in this study has been successfully applied to rapidly assemble multigene constructs (Engler and Marillonnet, 2014). Recently, new flexible gene cluster cloning workflows were proposed based on precise DNA cleavage tools (CRISPR system, especially near-PAMless SpCas9 variant SpRY) that can digest the target genome at specific sites even if there are no binding motifs of canonical restriction enzymes adjacent to bases of interest (Christie et al., 2022).

The antigenic polysaccharide synthesis gene clusters of target pathogens introduced into *E coli* host cells remained unchanged with their upstream sequences in most studies on developing bioconjugate vaccines using PGCT. This approach ensures the function of the exogenous genes but cannot regulate the synthesis of polysaccharide antigens. Another improvement in this study is replacing the original promoter with an inducible element, taking the first step forward in precisely controlling exogenous polysaccharides. With the increase in multi-omics data tools, especially quantitative transcriptomics (Ouyang et al., 2020), gene transcriptional regulatory elements, such as promotors and terminators, can be rationally designed and optimized for the target gene cluster. For example, various promoters with different activities (constitutive promoters or synthetic promoters) could be used to adjust the speed of polysaccharide antigen synthesis and coordinate with carrier protein and glycosyltransferase expression, thereby reducing the growth burden of the host cells and increasing the yield.

Most reports using PGCT are focused on carrier proteins used in licensed chemical conjugate vaccines. A different carrier protein rCTB was chosen in this study due to its ability to bind GM1, which is present on the surface of most cells, including macrophages and dendritic cells, to promote the antigen delivery process. The ability to form the correct pentamer (approximately 8 nm) is a prerequisite for CTB binding to GM1. We confirmed that protein glycosylation doesn’t alter the spatial structure of rCTB. Another advantage of multimeric protein carriers over monomeric carriers is that the antigenic polysaccharide can be intensively displayed and is, thus, easily recognized by antigen-presenting cells. Therefore, the characteristics and nanometer sizes of carrier proteins should be emphasized in the design of bioconjugate vaccines.

Host cell engineering is another crucial issue for PGCT technology. To avoid interference caused by endogenous LPS synthesis competing with recombinant glycans, K12-derived *E. coli* (deficient in O antigen synthesis due to mutation) is usually used as the host strain. Further modifications have been made to the basic strains to enhance the efficiency of protein glycosylation. The deletion of *waaL* (encoding the O-antigen ligase WaaL) (Feldman et al., 2005) and *wecA* (catalyzing initial transfer of N-acetylglucosamine on to the lipid carrier UndP) (Garcia-Quintanilla et al., 2014; Musumeci et al., 2014; Kay et al., 2022) is a widely accepted strategy. The double-mutant strains have been demonstrated to be suitable for the preparation of the bioconjugate vaccines against *F. tularensis* and *B. pseudomallei*. However, knocking out *wecA* isn’t suitable for the synthesis of all exogenous polysaccharides (Lehrer et al., 2007), such as the YeO9 OPS used in this experiment. The synthesis of OPS in YeO9 relies on an ABC transporter-dependent pathway, in which the GlcNAc initiating transferase WecA is required to add the primer monosaccharide to facilitate the following assembly of the adapter and repeat unit domain (Greenfield and Whitfield, 2012). Therefore, another host strain described recently (Peng et al., 2021) with the double deletions of *waaL* and *wbbH-L* gene clusters was used in this study, which might be a more suitable engineered strain for bioconjugate vaccine against *B. abortus*.

Macrophages are the main target cells for *Brucella* invasion and induce pro-inflammatory responses and the secretion of inflammatory factors (Baldi and Giambartolomei, 2013), such as TNF-α, IFN-γ, and IL-12 (Murphy et al., 2001). After infection with *B. abortus*, the level of TNF-α will continue to increase, leading to the production of IL-12 and IFN-γ (Dorneles et al., 2015). In this study, the levels of TNF-α, IFN-γ, and IL-12 in the serum of mice immunized with CTB-OPS_Ba_ remained lower compared to those in the control group, suggesting that vaccinated mice have pre-existing protective antibodies with good bactericidal activity. Two models of *Brucella* infection with different doses were *g*enerated to obtain a more comprehensive assessment of the protective effects of our bioconjugate vaccines. The sublethal challenge simulates the main characteristics of *Brucella* infection by causing spleen and liver tissue damage, such as mild lymphocyte depletion and macrophage infiltration in splenic nodules and liver granuloma production (Grillo et al., 2012). The lethal challenge is a common model for studying sepsis-causing bacteria and has been used in vaccine research to assess protection against various pathogens. The combination of two infection models can clearly provide more intuitive results for testing vaccine efficacy. It is satisfactory that both of these experiments showed that the bioconjugate vaccines produced here by engineered *E. coli* could effectively protect mice against bacterial infections, suggesting the potential for clinical application.

Our study provides a better strategy to address the safety issues in preparing classic *B. abortus* vaccines. Using engineered *E. coli* as a chassis to produce the bioconjugate vaccine against *B. abortus* by PGCT technology significantly reduces the risk of infection and allows for large-scale production. In the future, we will continue to optimize *Brucella* vaccine production *via* engineered *E. coli*. For example, protein antigens expressed by *E coli* host cells can be mixed with the polysaccharide antigens developed in this study, which will provide better protection due to improved cellular immunity.

## Data Availability

The original contributions presented in the study are included in the article/Supplementary Material, further inquiries can be directed to the corresponding authors.

## References

[B1] Baldi P. C. Giambartolomei G. H. (2013). Immunopathology of Brucella infection. Recent Pat. Antiinfect Drug Discov. 8, 18–26. 10.2174/1574891x11308010005 22812614

[B2] Beddoe T. Paton A. W. Le Nours J. Rossjohn J. Paton J. C. (2010). Structure, biological functions and applications of the AB5 toxins. Trends Biochem. Sci. 35, 411–418. 10.1016/j.tibs.2010.02.003 20202851 PMC2929601

[B3] Berti F. Adamo R. (2018). Antimicrobial glycoconjugate vaccines: An overview of classic and modern approaches for protein modification. Chem. Soc. Rev. 47, 9015–9025. 10.1039/c8cs00495a 30277489

[B4] Bundle D. R. Cherwonogrodzky J. W. Gidney M. A. Meikle P. J. Perry M. B. Peters T. (1989). Definition of Brucella A and M epitopes by monoclonal typing reagents and synthetic oligosaccharides. Infect. Immun. 57, 2829–2836. 10.1128/iai.57.9.2829-2836.1989 2474505 PMC313534

[B5] Christie K. A. Guo J. A. Silverstein R. A. Doll R. M. Mabuchi M. Stutzman H. E. (2022). Precise DNA cleavage using CRISPR-SpRYgests. Nat. Biotechnol. 10.1038/s41587-022-01492-y PMC10023266 36203014

[B6] De Figueiredo P. Ficht T. A. Rice-Ficht A. Rossetti C. A. Adams L. G. (2015). Pathogenesis and immunobiology of brucellosis: Review of brucella-host interactions. Am. J. Pathol. 185, 1505–1517. 10.1016/j.ajpath.2015.03.003 25892682 PMC4450313

[B7] Dorneles E. M. Teixeira-Carvalho A. Araujo M. S. Sriranganathan N. Lage A. P. (2015). Immune response triggered by Brucella abortus following infection or vaccination. Vaccine 33, 3659–3666. 10.1016/j.vaccine.2015.05.057 26048781

[B8] Dow J. M. Mauri M. Scott T. A. Wren B. W. (2020). Improving protein glycan coupling technology (PGCT) for glycoconjugate vaccine production. Expert Rev. Vaccines 19, 507–527. 10.1080/14760584.2020.1775077 32627609

[B9] Engler C. Marillonnet S. (2014). Golden gate cloning. Methods Mol. Biol. 1116, 119–131. 10.1007/978-1-62703-764-8_9 24395361

[B10] Feldman M. F. Wacker M. Hernandez M. Hitchen P. G. Marolda C. L. Kowarik M. (2005). Engineering N-linked protein glycosylation with diverse O antigen lipopolysaccharide structures in *Escherichia coli* . Proc. Natl. Acad. Sci. U. S. A. 102, 3016–3021. 10.1073/pnas.0500044102 15703289 PMC549450

[B11] Galindo C. L. Rosenzweig J. A. Kirtley M. L. Chopra A. K. (2011). Pathogenesis of Y. Enterocolitica and Y. Pseudotuberculosis in human yersiniosis. J. Pathog. 2011, 182051–182116. 10.4061/2011/182051 22567322 PMC3335670

[B12] Garcia-Quintanilla F. Iwashkiw J. A. Price N. L. Stratilo C. Feldman M. F. (2014). Production of a recombinant vaccine candidate against Burkholderia pseudomallei exploiting the bacterial N-glycosylation machinery. Front. Microbiol. 5, 381. 10.3389/fmicb.2014.00381 25120536 PMC4114197

[B13] Greenfield L. K. Whitfield C. (2012). Synthesis of lipopolysaccharide O-antigens by ABC transporter-dependent pathways. Carbohydr. Res. 356, 12–24. 10.1016/j.carres.2012.02.027 22475157

[B14] Grillo M. J. Blasco J. M. Gorvel J. P. Moriyon I. Moreno E. (2012). What have we learned from brucellosis in the mouse model? Vet. Res. 43, 29. 10.1186/1297-9716-43-29 22500859 PMC3410789

[B15] Harding C. M. Feldman M. F. (2019). Glycoengineering bioconjugate vaccines, therapeutics, and diagnostics in *E. coli* . Glycobiology 29, 519–529. 10.1093/glycob/cwz031 30989179 PMC6583762

[B16] Harding C. M. Nasr M. A. Scott N. E. Goyette-Desjardins G. Nothaft H. Mayer A. E. (2019). A platform for glycoengineering a polyvalent pneumococcal bioconjugate vaccine using *E. coli* as a host. Nat. Commun. 10, 891. 10.1038/s41467-019-08869-9 30792408 PMC6385209

[B17] Huang J. Pan C. Sun P. Feng E. Wu J. Zhu L. (2020). Application of an O-linked glycosylation system in Yersinia enterocolitica serotype O:9 to generate a new candidate vaccine against Brucella abortus. Microorganisms 8, 436. 10.3390/microorganisms8030436 32244903 PMC7143757

[B18] Iwashkiw J. A. Fentabil M. A. Faridmoayer A. Mills D. C. Peppler M. Czibener C. (2012). Exploiting the Campylobacter jejuni protein glycosylation system for glycoengineering vaccines and diagnostic tools directed against brucellosis. Microb. Cell Fact. 11, 13. 10.1186/1475-2859-11-13 22276812 PMC3298491

[B19] Kay E. J. Mauri M. Willcocks S. J. Scott T. A. Cuccui J. Wren B. W. (2022). Engineering a suite of *E. coli* strains for enhanced expression of bacterial polysaccharides and glycoconjugate vaccines. Microb. Cell Fact. 21, 66. 10.1186/s12934-022-01792-7 35449016 PMC9026721

[B20] Kay E. J. Yates L. E. Terra V. S. Cuccui J. Wren B. W. (2016). Recombinant expression of Streptococcus pneumoniae capsular polysaccharides in *Escherichia coli* . Open Biol. 6, 150243. 10.1098/rsob.150243 27110302 PMC4838161

[B21] Le Guern A. S. Martin L. Savin C. Carniel E. (2016). Yersiniosis in France: Overview and potential sources of infection. Int. J. Infect. Dis. 46, 1–7. 10.1016/j.ijid.2016.03.008 26987478

[B22] Lehrer J. Vigeant K. A. Tatar L. D. Valvano M. A. (2007). Functional characterization and membrane topology of *Escherichia coli* WecA, a sugar-phosphate transferase initiating the biosynthesis of enterobacterial common antigen and O-antigen lipopolysaccharide. J. Bacteriol. 189, 2618–2628. 10.1128/jb.01905-06 17237164 PMC1855806

[B23] Li X. Pan C. Liu Z. Sun P. Hua X. Feng E. (2022). Safety and immunogenicity of a new glycoengineered vaccine against Acinetobacter baumannii in mice. Microb. Biotechnol. 15, 703–716. 10.1111/1751-7915.13770 33755314 PMC8867989

[B24] Linton D. Dorrell N. Hitchen P. G. Amber S. Karlyshev A. V. Morris H. R. (2005). Functional analysis of the Campylobacter jejuni N-linked protein glycosylation pathway. Mol. Microbiol. 55, 1695–1703. 10.1111/j.1365-2958.2005.04519.x 15752194

[B25] Masjedian Jezi F. Razavi S. Mirnejad R. Zamani K. (2019). Immunogenic and protective antigens of Brucella as vaccine candidates. Comp. Immunol. Microbiol. Infect. Dis. 65, 29–36. 10.1016/j.cimid.2019.03.015 31300122

[B26] Mettu R. Chen C. Y. Wu C. Y. (2020). Synthetic carbohydrate-based vaccines: Challenges and opportunities. J. Biomed. Sci. 27, 9. 10.1186/s12929-019-0591-0 31900143 PMC6941340

[B27] Mirnejad R. Jazi F. M. Mostafaei S. Sedighi M. (2017). Epidemiology of brucellosis in Iran: A comprehensive systematic review and meta-analysis study. Microb. Pathog. 109, 239–247. 10.1016/j.micpath.2017.06.005 28602839

[B28] Morais V. Suarez N. (2022). Conjugation mechanism for pneumococcal glycoconjugate vaccines: Classic and emerging methods. Bioeng. (Basel) 9 (12), 774. 10.3390/bioengineering9120774 PMC9774679 36550980

[B29] Murphy E. A. Parent M. Sathiyaseelan J. Jiang X. Baldwin C. L. (2001). Immune control of Brucella abortus 2308 infections in BALB/c mice. FEMS Immunol. Med. Microbiol. 32, 85–88. 10.1111/j.1574-695x.2001.tb00536.x 11750226

[B30] Musumeci M. A. Faridmoayer A. Watanabe Y. Feldman M. F. (2014). Evaluating the role of conserved amino acids in bacterial O-oligosaccharyltransferases by *in vivo*, *in vitro* and limited proteolysis assays. Glycobiology 24, 39–50. 10.1093/glycob/cwt087 24092836

[B31] Nothaft H. Szymanski C. M. (2010). Protein glycosylation in bacteria: Sweeter than ever. Nat. Rev. Microbiol. 8, 765–778. 10.1038/nrmicro2383 20948550

[B32] Ouyang Q. Wang X. Zhang N. Zhong L. Liu J. Ding X. (2020). Promoter screening facilitates heterologous production of complex secondary metabolites in burkholderiales strains. ACS Synth. Biol. 9, 457–460. 10.1021/acssynbio.9b00459 31999442

[B33] Pan C. Sun P. Liu B. Liang H. Peng Z. Dong Y. (2016). Biosynthesis of conjugate vaccines using an O-linked glycosylation system. mBio 7, e00443–e00416. 10.1128/mbio.00443-16 27118590 PMC4850263

[B34] Peng Z. Wu J. Wang K. Li X. Sun P. Zhang L. (2021). Production of a promising biosynthetic self-assembled nanoconjugate vaccine against Klebsiella pneumoniae serotype O2 in a general Escherichia coli host. Adv. Sci. (Weinh) 8, e2100549. 10.1002/advs.202100549 34032027 PMC8292882

[B35] Rivas L. Strydom H. Paine S. Wang J. Wright J. (2021). Yersiniosis in New Zealand. Pathogens 10 (2), 191. 10.3390/pathogens10020191 33578727 PMC7916520

[B36] Skurnik M. Biedzka-Sarek M. Lubeck P. S. Blom T. Bengoechea J. A. Perez-Gutierrez C. (2007). Characterization and biological role of the O-polysaccharide gene cluster of Yersinia enterocolitica serotype O:9. J. Bacteriol. 189, 7244–7253. 10.1128/jb.00605-07 17693522 PMC2168460

[B37] Sun P. Pan C. Zeng M. Liu B. Liang H. Wang D. (2018). Design and production of conjugate vaccines against S. Paratyphi A using an O-linked glycosylation system *in vivo* . NPJ Vaccines 3, 4. 10.1038/s41541-017-0037-1 29423317 PMC5799188

[B38] Von Bargen K. Gorvel J. P. Salcedo S. P. (2012). Internal affairs: Investigating the Brucella intracellular lifestyle. FEMS Microbiol. Rev. 36, 533–562. 10.1111/j.1574-6976.2012.00334.x 22373010

