# Supplementary material for: A bioconjugate vaccine against Brucella abortus produced by engineered Escherichia coli
Source: Front Bioeng Biotechnol. 2023 Feb 23;11:1121074. doi: 10.3389/fbioe.2023.1121074 (PMC9995886; doi:10.3389/fbioe.2023.1121074)
Supplement: Supplementary file 1 [file DataSheet1.docx]

Supplementary Material

**A bioconjugate vaccine against *Brucella abortus* produced by engineered *Escherichia coli***

Shulei Li, Jing Huang, Kangfeng Wang, Yan Liu, Yan Guo, Xiang Li, Jun Wu, Peng Sun, Yufei Wang*, Li Zhu*, Hengliang Wang*

Shulei Li and Jing Huang contributed equally to this work

*** Correspondence:**

Hengliang Wang (wanghl@bmi.ac.cn)

Li Zhu (jewly54@bmi.ac.cn)

Yufei Wang (tosya@163.com)

## 1. Supplementary Figures


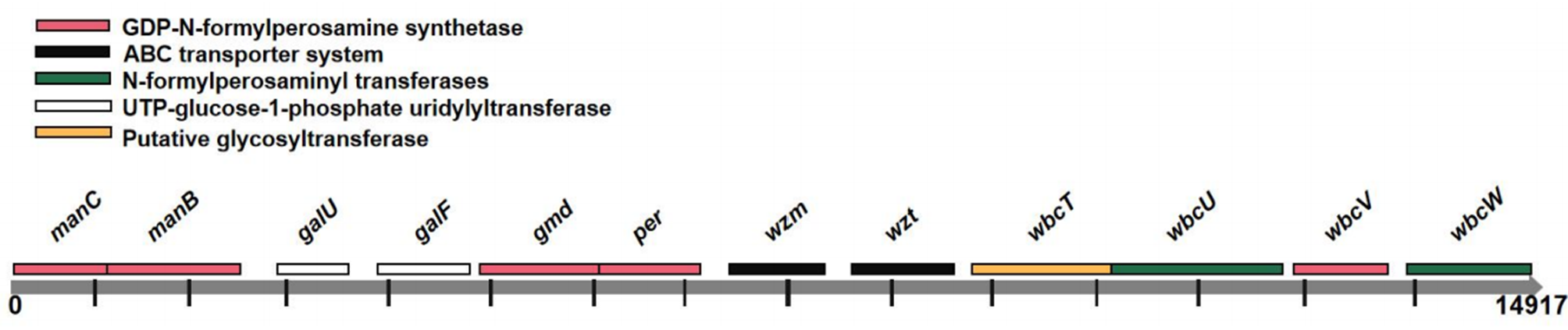


**Supplementary Figure 1** YeO9 O-polysaccharide gene cluster and functional region.


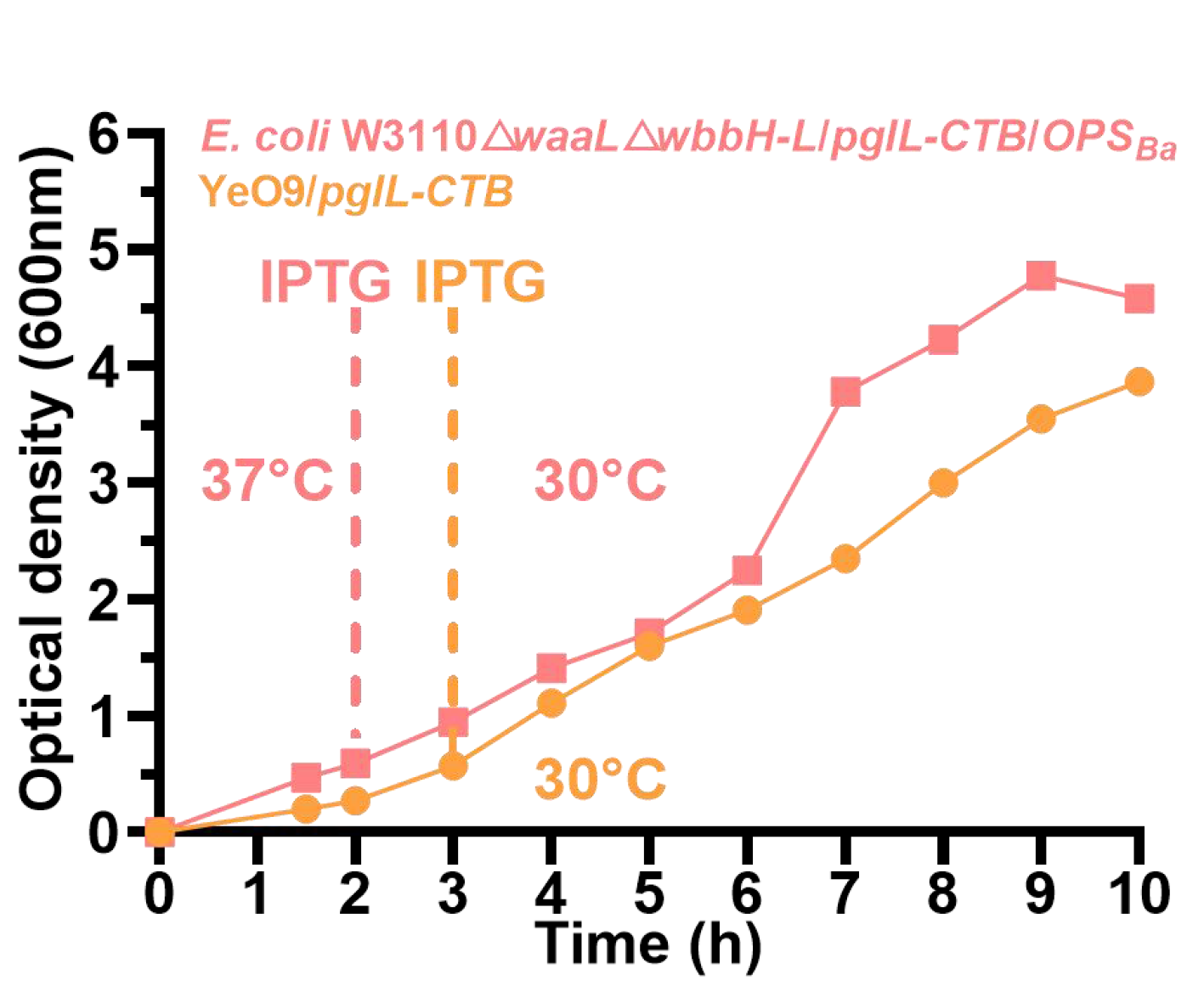


**Supplementary Figure 2.** Growth curves of different host organisms for the production of *Brucella* bioconjugate vaccine.


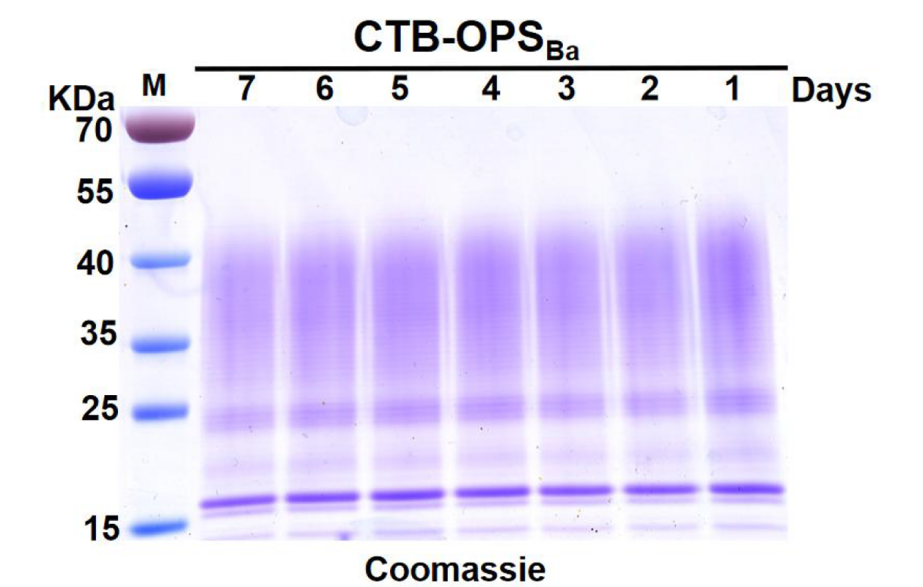


**Supplementary Figure 3.** Coomassie blue staining of CTB-OPS_Ba_ stored at room for 7 days.


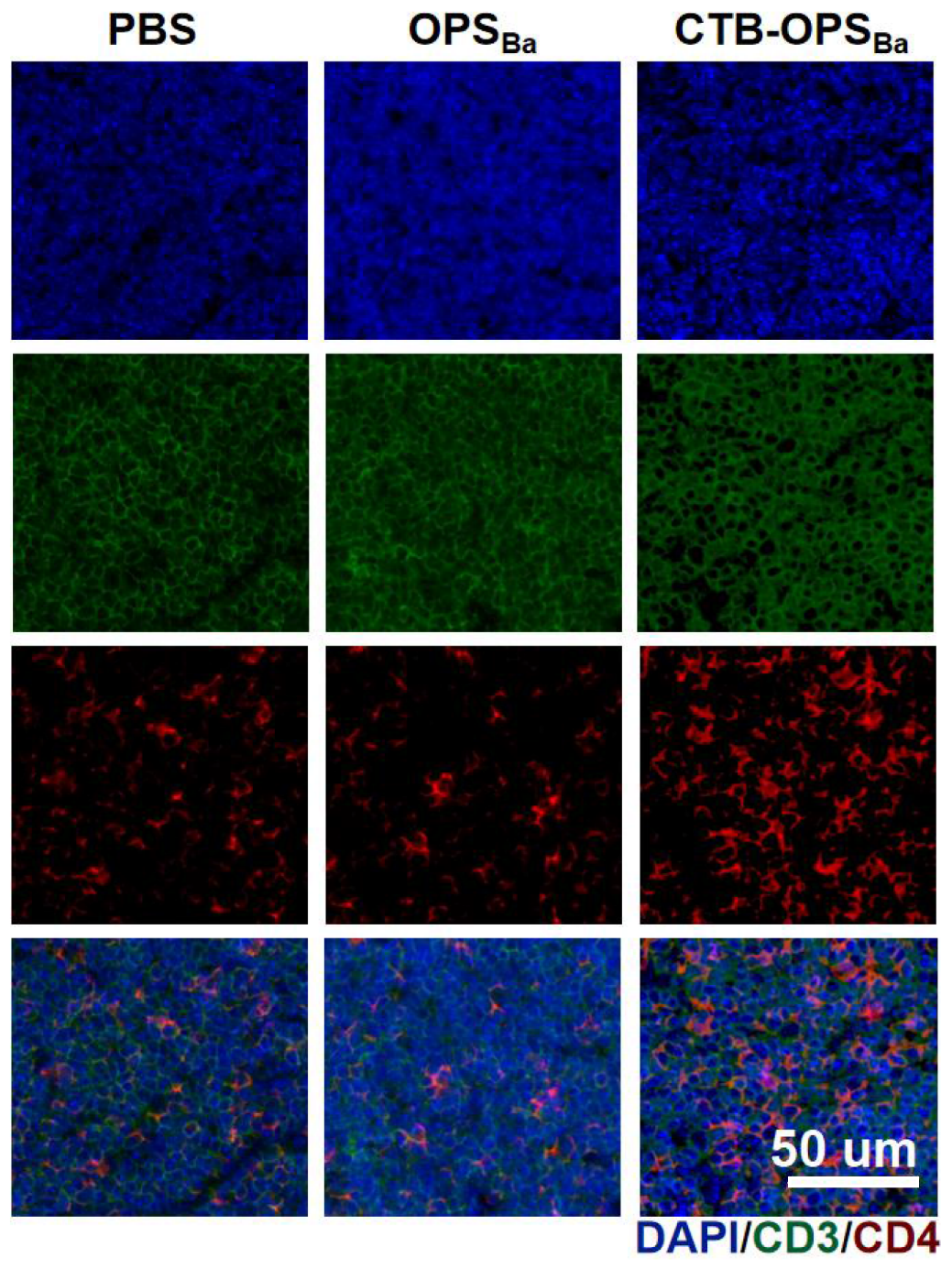


**Supplementary Figure 4.** CD3^+^ and CD4^+^ cells in the spleen were detected by immunofluorescence.


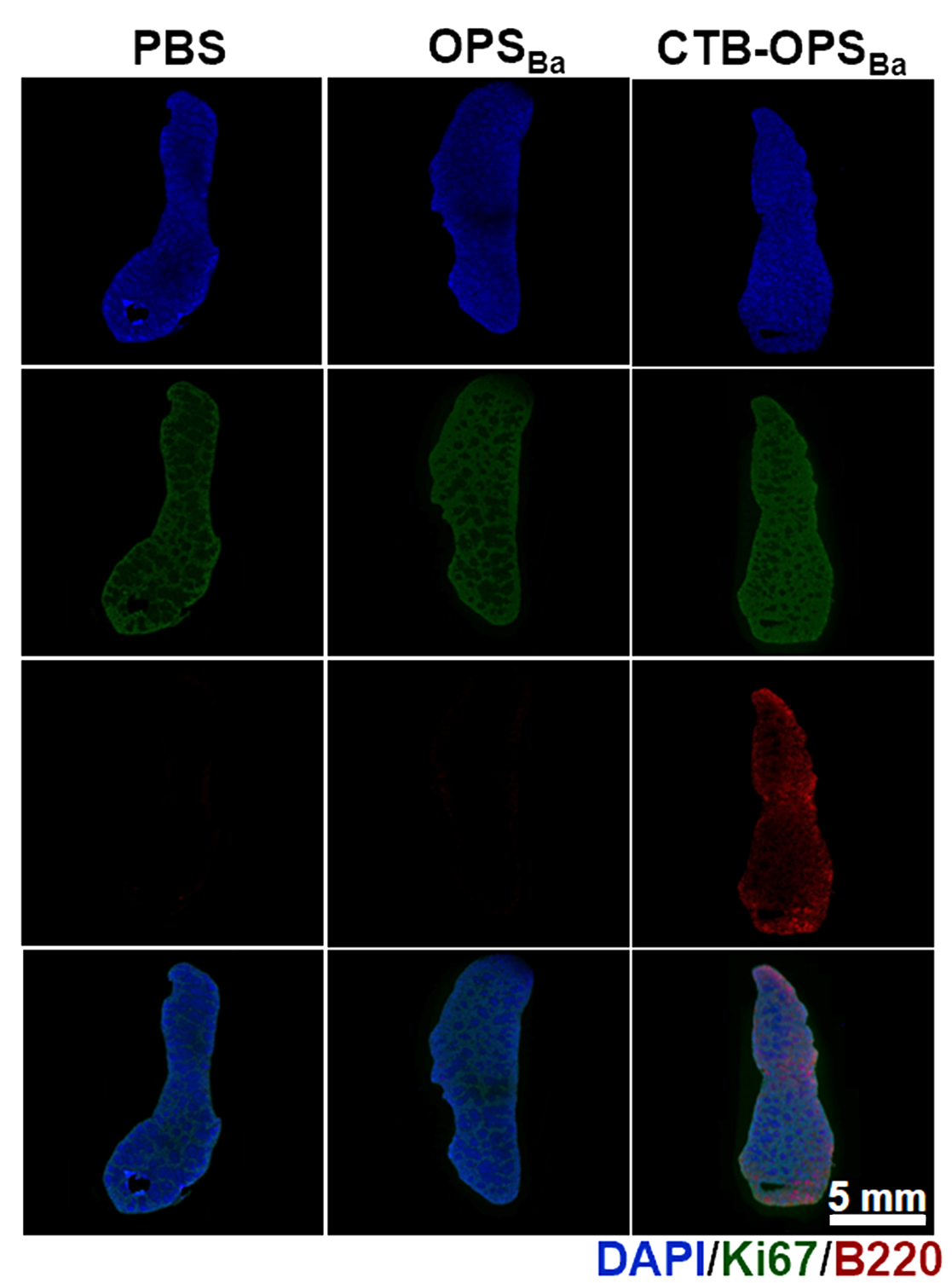


**Supplementary Figure 5.** Ki67 and B220 in the spleen were detected by immunofluorescence.


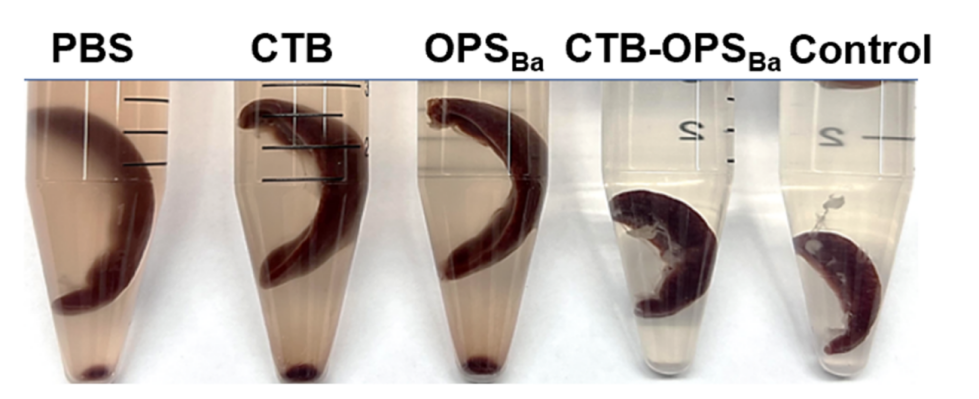


**Supplementary Figure 6.** Images of mouse spleens 7 days after infection with a non-lethal dose of *B.abortus* A19.

**2. Supplementary Tables**

**Supplementary Table 1** Bacterial strains and plasmids used in this study.

| **Bacterial Strains and Plasmids** | **Characteristic** | **Source** |
| --- | --- | --- |
| *B.abortus* A19  *Y. enterocolitica* O:9 (YeO9) | Attenuated *Brucella abortus*  O antigen structure similar to *B. abortus* | Institute for Communicable Disease Prevention, Chinese Centre for Disease Control and Prevention |
| *E. coli* W3110 | Gene *wbbL* was inactivated | Laboratory stock |
| *E.coli* W3110△*waaL*△*wbbH-L* | Absence of *waal*, *wbbH*, *wbbJ*, *wbbK*, *wbbL* genes | Laboratory stock |
| *E.coli* W3110/pACYC184tac-OPS_Ba_ | Heterologous synthesis of YeO9 OPS | This study |
| *E. coli* W3110△*waaL*△*wbbH-L/*pET28a-*pglL-CTB* pACYC184tac-OPS_Ba_ | Expression of glycoprotein  CTB-OPS_Ba_ | This study |
| pACYC184tac | Containing Tac promoter, lac operater, and ribosome binding site, Cm^r^ | This study |
| pET28a-*pglL*-*CTB* | Encodes PgIL and 6×His-tagged CTB fused DsbA signal peptide at N-terminus and glycosylation sequence (4573) fragment at C-terminus, Kan^r^ | Laboratory stock |
| pACYC184tac-OPS_Ba_ | Encodes O-polysaccharide of YeO9, Cm^r^ | This study |

**Supplementary Table 2** All primers used to construct and confirm gene mutants in this study.

| **Primer** | **Sequence** |
| --- | --- |
| pACYC184 F | ACCAggTCTCCgATgAACggTTCTggCAAATATTCTgAA |
| pACYC184 R | ACCAggTCTCCgCTAgAATTCTgTTTCCTgTgTg |
| tac-RBS F | ACCAggTCTCCCATCgTTTCCTgTgTgAAATTgTTATC |
| tac-RBS R | ACCAggTCTCCTAgCAgAATTCTAgCTCTgAgTTAC |
| pACYC184tac F | ACCAggTCTCCTAgCTAgCTCTgAgTTACAACAgTCC |
| pACYC184tac R | ACCAggTCTCCCATCgAATTCTgTTTCCTgTgTgAAATTg |
| manC-manB F | ACCAggTCTCCgATgATgTTACTTCCggTgATTA |
| manC-manB R | ACCAggTCTCCgCCTCTAATTACTAgACCTTATTTCTT |
| galu-gmd F | ACCAggTCTCCAggCATgAAATgTTTgAAAgCAgTCATTCCT |
| galu-gmd R | ACCAggTCTCCggCATTATTCAATAgAAACACTAACATCAT |
| per-wzt F | ACCAggTCTCCTgCCATgATAATACCAATATATCAgCCT |
| per-wzt R | ACCAggTCTCCAgTgTTAATTTAATATTTCATTAACTTTTTTAAATT |
| wbct-wbcu F | ACCAggTCTCCCACTATgAgCgATAACAAAAgAATTTTATggT |
| wbct-wbcu R | ACCAggTCTCCCgACTCATTCCACAgTTAATACATCAATATA |
| wbcV-wbcW F | ACCAggTCTCCgTCgATgATTCTTATgAATATAgAAAATAAAAg |
| wbcV-wbcW R | ACCAggTCTCCgCTATCACTCTAAAAgAgATCTATATATT |

All plasmid constructs were generated using the Golden Gate Assembly Kit (BsaI-HFv2).
